# The Secret of the Servant Girl Trouble

**Published:** 1882-07

**Authors:** Ben. H. Pratt


### THE BISTOURY.

Vol. ~~XIX~~. XVIII    ELMIRA, N. Y., JULY, 1882.    No. 2.

### Contributions.

### THE SECRET OF THE SERVANT GIRL TROUBLE.

BEN. H. PRATT.

"I want my girls to learn how to do every kind of housework, not because I expect they will ever have to do it, but every woman ought to know how it's done, in order to properly superintend the affairs of her own household." That's about the formula, more or less varied, which we have all heard time and again as one has sat reading his evening paper while his wife and his neighbor's wife discuss domestic affairs the evening through at the other end of the center-table. Of course no man cares to throw down his paper—even if he hasn't read a word for the last hour and a half—to enter into a combat of words against such tremendous odds as the situation indicates; nor does he feel like meeting the first argumentative slap in the shape of "what do you men know about these things, anyway? You never kept house; you never managed a servant girl; you don't know any more about running a domestic establishment than a child."

Perhaps not. But it isn't necessary that one should know all about conducting a household, in order to see what follies are tolerated therein under the guise of mistaken principles. Now, it's all bosh to claim that, "to know how" is the desired, or rather desirable, aim of the mother in teaching her girls the theory and practice of housekeeping. They should be taught these, that they may not only know how to work, and thus intelligently direct servants in the performance of household duties, but to take hold and do that same work when it isn't absolutely necessary. Housework is woman's natural occupation. It's her business, and should be made her recreation as well. Men, whose business isn't their recreation, become the unhappiest of mortals. Women, without occupation—and nine-tenths of them have no real occupation besides their household duties—are the stupidest and most pitiable creatures on earth. I don't care how much of a woman's time is taken up in social duties—in making and receiving calls—in visitings and entertainings—in benevolent work—in missionary enterprises—in reading and writing—in fancy work for pleasure merely—in any of the hundred odd schemes which the age has invented to keep a woman employed—so long as somebody else does all of her legitimate work for her she is simply an object of pity. Nobody can fold their hands and sit down and do nothing, and hence it is that woman's brain is ever occupied with inventing some pleasing way of spending time that she ought not to have to spend in that way. It is the too prevalent desire on the part of the men to relieve their wives of everything that can possibly be construed into a semblance of real work, that drives women into such nonsensical dressing—into such labyrinths of fancy work—into such tastes for trashy reading—into such extravagant notions about so-called charitable work—into social unhappinesses of every sort—into mopery and languidity and uselessness. One cannot make of life one eternal round of pleasure—a constant doing of only those things which are intended to be only occasional and recreational. One palls of the enjoyable much sooner than of the practical and helpful. A certain amount of genuine labor is necessary to human happiness. We must have a quantum of drudgery in this world in order to properly enjoy occasional seasons of repose and relaxation. Constant repose or relaxation takes away all capacity for enjoyment. Work and play must be *mixed* in this life to give it any zest, and there must be a good deal more work than play, too.

Now, then, girls should be early apprenticed to good housekeepers and kept at the trade not only until they learn it well, but like it thoroughly. Most girls are taught domestic duties much as they are drilled in the branches pursued in their schools. They go through the routine from a sense of duty, or obedience, measuring off so much of acquirement from day to day, month to month and year to year, looking eagerly forward to the time when mother will say, "now you know all about housework, and you needn't bother over it any more. I'll take your chances on knowing how to conduct your future establishment when the time comes. Now you may give your attention to your school or your dress, or your friends or your music, as the case may be," and then the daughter begins to pose for the man that's to set her up in that home, which is seldom absent from the visions of the average young woman. She graduates in housekeeping just as she does from boarding school, happy that she's learned it all and hasn't got to study or worry any more. And she drops her housework as she does her French, and her mathematics, and her history, having mastered the theory, and hoping she may never be compelled to bring it into practice again. She *can* cook and scrub, and sweep and sew, but never means to do it again unless compelled. When such are obliged, by fate, to resume their long abandoned domestic accomplishments, how few there are who can revive them to any purpose. How few such make school teachers or music teachers of any account, or can manage a home without that modern curse, a hired girl!

As there is none so rich that she can endure life without occupation, so there is none so competent to hire all her housework done, that she can afford to be the mere figure-head of an establishment. She who thinks that she now has nothing to do but enjoy life, soon finds herself the unhappiest of women. She may now and then inspire a semblance of respect from her help, because of the glimpse she occasionally reveals that she knows how things ought to be done, but it isn't a tithe of that regard, approaching close upon veneration, which she evokes who combines with her, "know how" the actual taking hold and demonstrating her willingness to, even her love for, work—putting her own hands to it with that zest which ever accompanies real, personal labor, manual and—in one sense, though not the perverted one—menial. She is the happiest queen of the household, who cannot be compromised by going into her own kitchen and sharing the labors therein, if she feels like it, or occasion demands it, with the hired help. Then it is that the woman truly reigns in her own household, its very queen, silently asserting her independence and giving it quietly out that she's not at the mercy of her servant, and come what of insubordination there may, there's always a hand ready to seize the helm and conduct the home craft through no end of squalls.

I know a lady who thoroughly understands the theory and practice of housekeeping and housework. She keeps a number of servants, a cook, a chambermaid, a laundress, a nursemaid. But there is no manner of work done within her household that she cannot and does not occasionally do herself, sometimes working with her help, sometimes by herself. The hired man of the family had always had his washing and ironing done in the house. It happened upon a time that her laundress was new to the place, and judging from the number of servants about, very reasonably concluded that the mistress didn't even superintend the help, much less do anything herself. When the hired man's clothes were brought to this washer the first Monday morning, and she observed how grimy they were, she at once rebelled against washing them, and remarked that madam couldn't think much of her help to give them such dirty work to do. The expression was conveyed to the ears of madam, who at once proceeded to the laundry, bade the dainty washer to merely wait upon her and she'd wash the objectionable garments herself. She did so, exhibiting not only skill but strength therefor, and accomplished the work easily and smilingly. But she purposely required the shirking woman to bring her so much water, and so many utensils, and made such a mess upon the floor, and used so many vessels which required cleaning up, that the complainer made up her mind that it would have been far less trouble, and much more agreeable, to have washed the clothes herself than wait upon madam. Therefore, when the latter informed the now rather subdued washer that hereafter, when she came to the hired man's clothing she should always send for her mistress, who would cheerfully do the job each week, the girl made up her mind that this was no woman to be trifled with or bullied into any false consideration for her help, and thereafter she did the entire washing herself without making any demurrer.

I repeat, not merely to know how, should girls learn to do all manner of domestic routine work, but to continually practice because it is a necessity to their well being and well feeling, and constitutes a portion of their life which they may not escape from without suffering the penalty thereof. It is a woman's never-failing remedy for that ennui which her indoor life and often long protracted periods of being alone subject her to. Even society, no matter how frequent its calls upon her for contributions of work and talk and expedient to kill time, becomes monotonous, and it is as necessary that she have just the escapement which housework affords to keep her in good mental and physical balance, and enable her to sustain her relations to the world, the family and herself, which go to complete a well-rounded life. There are so many women that are angularized as it were—cornered into sharpness on one side and dull flatness on the other—women not done clear through—bright and breezy and fresh to day, and down stupid with the dumps to-morrow—women who require a novelty or a temporary excitement to make them endurable. Watch these, and see if they are not of those who, having no fixed, certain, every-day occupation to make their lives complete, with just the proper quantum of in-chinking of play and recreation, are more than half the time wondering what they shall hit upon for their next means of entertainment. They don't realize that entertainment, sought after for itself alone, is the dreariest experience in life. It's like a diet all cake. Cake is good, but you can't enjoy cake three times a day. Work, not worsted, not crochet, not plaque-painting, not fancy work of any kind, but useful, help-somebody-along sort of work, that sets you thinking and walking and lifting and pushing and reaching and panting even, and has large variety about it—in a word, old-fashioned housework; not to save, or economize, or because you can do it better or as well as your hired girl, but because it's a good thing to spread over every day of your life to keep you in condition to be jolly when the time comes for you to be so. The girl that despises work is a fool. She's a fool because she doesn't know what's the best thing for her. She thinks that because cake and peaches and cream are good, she can't have too much of them. Let her try these for steady awhile, and she'll experience precisely the same feelings, physically, that pleasure alone will bring to her mental and moral economy. The woman that can't work is an object of pity beyond any living thing except the woman that won't work because she "doesn't have to."

But there's another need for woman's work than her own selfish well being. By assuming her own place, and the duties which her sphere rightly inherits, she holds in her own hand the happiness of every member of her household. If she delegate these duties to another and allow a hireling to fill the position that she should religiously and conscientiously occupy, she cannot wholly rule in her domestic realm. Help, the best of its sort, is not absolutely reliable. It cannot, even if it would, identify itself so thoroughly with the needs, the comforts, the domesticity of the home, that the controlling and regulating element of the household economy shall not be found lacking somewhere. She who rules but by proxy in her own domain is liable to have unhappy, if not rebellious subjects. There is no home that can be committed to the care of "help" alone, or even to that element under strict supervision, if such supervision be of the head only. She who is too nice to soil her hands occasionally, and is fully satisfied with exercising the theory of home governing, will find to her chagrin, if not to her sorrow, that she has made a fatal mistake, and should early take such steps as may remedy it.

It is not so much the ignorance of mistresses as their distate for routine household duties that gives society so much trouble with its help. Hired female servants are characterized, as a class, as independent minxes whom one cannot reprimand, however mildly, or even advise, without their getting into a pet and deserting their places. But their independence is not to be wondered at. They usually know as much about their business as their mistresses, and because of the latter's lack of practice have far better methods of accomplishing their work than those who employ them. It isn't any wonder that skill will not brook dictation from mere bungling, and so the servant holds her place no longer than she can do so untrammeled by crotchety dictation. The largest share of our help-employers, however well they may have been trained to household government by their mothers before them, having been trained under the learn-to-know-how rather than the learn-to-actually-do principle, are incompetent to manage help. Seldom, if ever working themselves, they cannot sympathize with the worker, or put themselves in her place. He who has never been a private soldier cannot become an effective commander of troops. The respect which a working mistress inspires within the breast of a servant goes far to make the servant faithful and loving. No servant can well serve one whom she does not, in some measure, love. Mistresses are oftener pitied for their ignorance or laziness than loved for their kindness or consideration. The pitied mistress is scarcely more to be envied than the hated one. Unless there be mutual and genuine regard between misstress and maid there will be eternal clashings, if not speedy separations. The mistress who has won the love of a servant has achieved much in the direction of a smoothly conducted household. Fear of a mistress cannot be relied upon a moment. No servant worth having can be influenced by it out of the sight of her whom she fears.

But to cut short an article already too prolix, and condense into a sentence my object in the foregoing,—I claim that the woman who does not, except at a pinch, under protest, or driven by necessity stand at the wash-tub, or over the range, or at the pastry board *with* her help, is sure to be one of those mistresses who is continually wondering why she can't keep her help. Of course she can't, and she doesn't deserve to.

